# The Role of Entrepreneur Cognition on Core Rigidity

**DOI:** 10.3389/fpsyg.2021.717253

**Published:** 2022-02-02

**Authors:** Yan Guo, Pei-Wen Huang, Meihui Chou, Shih-Chieh Fang, Fu-Sheng Tsai

**Affiliations:** ^1^Department of Accounting and Financial Management, College of Management and Economics, Tianjin University, Tianjin, China; ^2^Department of Business Management, College of Management, Cheng Shiu University, Kaohsiung, Taiwan; ^3^Department of Business Administration, National Cheng Kung University, Tainan, Taiwan; ^4^North China University of Water Resources and Electric Power (NCWREP), Zhengzhou, China; ^5^Center for Environmental Toxin and Emerging-Contaminant Research, Cheng Shiu University, Kaohsiung, Taiwan; ^6^Super Micro Mass Research and Technology Center, Cheng Shiu University, Kaohsiung, Taiwan

**Keywords:** WS Co., core capacity, core rigidity, cognition of managers, entrepreneur

## Abstract

Against the backdrop of economic internationalization and market globalization, the world has witnessed faster competitive contents with a more dynamic market environment, a more rapid technological innovation, and more diverse customer needs. Thus, for every enterprise especially led by entrepreneurs, the focus is to maintain the sustainability of competitive advantages and dynamically transform core capacity to avoid rigidity. This paper introduces the process of the deepened rigidity in WS Co. Company, which occurs due to the wrong cognition of Dr. S and his teams who managed or failed to respond to the rigidity encountered by the company during different periods. The rigidity of its capacity is believed to be caused by the cognitive errors of the entrepreneur and his team facing every period of potential rigidity, which makes them fail to deal with it (or choose the wrong methods), thus leading to complete rigidity. This paper intends to explain how rigidity takes shape from the cognitive aspect of senior managers, which may serve as illuminations for the similar enterprises led by entrepreneurs.

## Introduction

Since the 1990s, the theory of core capacity has aroused general concerns in the field of management, and core capacity has become the source of sustainable competitive advantage for enterprises. The current social environment is extremely dynamic, which is characterized by a rapid development in technology, the expansion of competition, the diversification of customer preferences, higher speed of information processing, etc. Facing the changes in their environment, a lot of enterprises still stick to their core competency in the long run, which thus leads to the rigidity of core capacity (hereinafter referred to as core rigidity) and failure. Explaining the cause of core rigidity thus becomes a problem to be solved during sustainable development for today’s enterprises. In China, especially for private enterprises, the situation of “manager-oriented” has already taken a shape, where the sustainable development of enterprises largely depends on the cognition and competence of managers. Thus, the influence of manager’s cognition in this process should not be underestimated when studying the cause of the gradual formation of core rigidity in enterprises.

In the late 1990s, core capacity plays a vital role in the research and practice of enterprise strategic management, which not only explores a new area for the theory of strategic management, but also leads a lot of enterprises to succeed in an increasingly fierce competition. Prahalad and Hamel (1990) first proposed the idea of core capacity, which were considered as the accumulation of knowledge within the organization, especially the knowledge about how to coordinate a variety of production skills and integrate the knowledge of different technologies. As core capacity is often characterized by its flexible and non-imitative qualities and a high user value, it forms the source of sustainable competitive advantage for enterprises (Prahalad and Hamel, 1990). One of the important issues in the theory of core capacity is the rigidity of core capacity. Leonard-Barton (1992) first studied the rigidity of core capacity. He thinks that people often exaggerate their success in the past, which often goes too far and thus hinders the development of the enterprise. Such an exaggeration is often reflected by overconfidence and overemphasis on goals. Companies that show a sign of rigidity often place a huge value on the problems in the existing market or those related to operations, thinking that there is no difference between the present and the future (Leonard-Barton, 1992). Core rigidity does not occur overnight. Instead, it can be seen from its definition that before it comes into being it is often inexplicit and has a latent period. Thus, it is of utmost importance to deal with potential rigidity.

Cognition is the basis for the development of organizational capabilities, especially for enterprises led by entrepreneurs (such as WS Co.). Thus, cognition may affect the process of capacity development, especially the cognition of how to deal with potential rigidity. Managers’ cognition has been increasingly regarded as a reasonable field of theoretical construction and an empirical study for strategic management, which has aroused the attention of a lot of strategic managers and led to the emergence of a large number of researches centered in managers’ cognition. In existing researches, scholars have proposed two dimensions to understand managers’ cognition – cognitive structure and cognitive process. From the perspective of cognitive structure, manager cognition is the belief or mental model that a strategic decision-maker in the enterprise has, which reflects the state of the external environment, strategy, business portfolio, or the organization (Thomas and Porac, 2002). From the perspective of cognitive process, manager cognition is the information processing for strategic decision-makers to understand the objective environment (Maynard, 1983). Then, will managers’ cognition lead to the rigidity of core capacity? If it does, through what way does it affect the rigidity of core capacity? Through a case analysis, this thesis points out the cognitive errors of the entrepreneur and his team facing every period of potential rigidity, which makes them fail to deal with it (or choose the wrong methods), thus leading to the final rigidity of the competence.

## Literature Review

### Core Capacity

From the late 1980s to early 1990s, the resource-based theory has begun to take a leading role in the field of strategic management, which considers the internal resources and capabilities of the enterprise as the source of its competitive advantage. In essence, the resource-based theory emphasizes that resources and capabilities are the genes of competitive advantage. As resources are heterogeneously distributed in an enterprise, valuable resources and capabilities are thus the sources of competitive advantage, and if such resources are difficult to replicate and replace, they become the source of sustainable competitive advantage. Yet, resource advantages are not enough. The enterprise must have unique ability to take advantage of these resources. The proposal of the idea on core capacity in fact marks the deepening and perfection of the research on the resource-based theory.

Prahalad and Hamel (1990) first proposed the idea of core capacity. They suggest that core capacity is the specific expertise studied and accumulated by the enterprise though the continuation and perfection of products and technology (Prahalad and Hamel, 1990). Chinese scholar Yi proves the value of core capacity to an enterprise through empirical research, which implies that core capacity is a prerequisite for an enterprise to gain and maintain its competitive advantage, as well as the key to its sustainable development and profit-making. Meanwhile, the same author divides the core capacity into three aspects – strategy, organization, and technology (Helfat and Peteraf, 2015).

### Capability Paradox

Considering that core capacity is rather static, it in fact reflects a “paradox”: the more emphasis the enterprise lays on core capacity, the stronger it is, which at last leads the enterprise to a decline or even a failure. “Extremity is deadening” best describes an enterprise’s core capacity. When the core capacity of an enterprise develops to an extreme level, deep rigidity is formed, which will then hinder the reforms within the enterprise, meanwhile restricting the renewal and reconstruction of its core capacity. Any changes in the external environment will greatly affect the enterprise’s business performance. Raised the three forms of capability paradox – path dependence, structural inertia, and commitment (Schreyögg and Kliesch-Eberl, 2010).

*Path dependence* means that once an enterprise chooses a system or technology, it will continue to develop toward the established direction and gain strength due to factors, including scale economy, coordination effect, learning effect, adaptive expectations, and vested interests. Path dependence gives the enterprise the process advantage that is hard for other enterprises to imitate, while through constant positive feedbacks it leads the enterprise to be restrained in its original successful experiences and modes, thus depriving it of the flexibility facing the changing environment. The enterprise’s overadherence to its strategic position is exactly due to the path dependence in the activities and capabilities of the organization. Once the present and future capabilities of an enterprise are determined by a “historical event,” due to the increasing revenue, self-reinforcing process will immediately take place when a positive feedback loop is formed by a series of successful activities.

*Structural inertia* refers to the lack of adaptability to change in an enterprise when it fails to break original behavior patterns and capacity structure. In other words, after operating for a while, an organization or a system tends to operate along the original path when the effect of external forces is removed. Structural inertia mainly indicates the inertia of the organization in its structure, policy, and management philosophy. Hannan and Freeman enumerated the four endogenous variables that play a key role in the form and structure of a stable organization: investment in a certain field in the past; the restraining effect of equipment, personnel, and information communication channels on the decision-makers; internal policies and the history of the organization; and the procedures and norms formed within the organization. Three external factors also restrain the changes in the form of the organization: obstacles to the market, external restraining of available information, and social pressure. The changes of these factors lead to the worst performance of the organization as the resources spent on production and competition reaction are replaced by high cost reorganization, which makes the structural inertia cost to succeed in the organization.

*Commitment* refers to the consequences of restricting organizational behavior incurred by any decision made by an enterprise. This commitment is both economical and psychological and tends to escalate with the increase of investment. Resource commitment is in fact an enterprise’s permission to allocate and use its own resources. The resources here refer to all tangible, intangible, static, and dynamic assets that the enterprise owns and can control, including funds, materials, manpower, information, technology, knowledge, capabilities, and reputation. Though these assets themselves will not produce economic benefits, they are the foundation for transformation into the final product or services. And resource commitment is that the enterprise agrees to make full use of and optimize the allocation of these resources, aiming at maximizing their profits, which emphasizes the importance of managers in the enterprise.

### Core Rigidity

As Prahalad and Hamel (1990) first proposed the idea of “core capacity,” “cultivating the enterprise’s core capacity and maintaining its sustainability” have become the key weapons adopted by a lot of enterprises to win advantage over their competitors. Some enterprises have even regarded it as the panacea for business operations. Yet, the commercial circle was too enthralled by this concept to realize that “extremity is always deadening.” It can be seen from the development process of a lot of well-known enterprises that they achieved a great success due to certain core capacity but often gradually declined if they stuck to such core capacity in the long run. The most successful enterprises are often least sensitive to changes in the external environment. Thus, the theory of core rigidity has emerged.

Leonard-Barton (1992) first raised the idea of core rigidity: owing to the long-term accumulation of core capacity, a kind of inertia is produced within the enterprise, making it hard to adapt to changes, which is called core rigidity. The overstability is caused by the products, market, technology, talent, and management that obtain success due to core capacity. To some extent, an objective existence is hard to be overcome by the enterprise itself, which can make the core capacity rigid and difficult to expand, thus restraining the continuous innovation of the enterprise (Leonard-Barton, 1992). Core rigidity and core capacity are the two sides of a coin. Lots of behaviors can cause rigidity in an enterprise, but only the rigidity caused by core capacity can be called core rigidity.

Believes that core rigidity means when the environment changes and new capabilities need to be developed in the organization, old knowledge and the use of original capabilities will hinder the efforts of enterprises to change their core competency (Levinthal and March, 1993). Bettis and Prahalad (1995) consider the leading logic as the restriction for seeking new business opportunities, which leads to core rigidity. Teece et al. (1997) think that successful competition is based on the enterprise’s ability to rebuild skills and resources of internal and external organizations in response to the dynamic environment. Further perfects the concept of core rigidity, regarding the excessive adherence to internal customs, habits, or practices of an enterprise as the essence of this concept. These customs, habits, or practices are the most difficult ones to change within an enterprise, and enterprise practice is in fact a phenomenon of path dependence.

### Treatment for Potential Rigidity

Core rigidity does not occur overnight. Instead, it can be seen from its definition that before it comes into being it is often inexplicit and has latent periods. Thus, it is of utmost importance to deal with potential rigidity. Schreyögg and Kliesch-Eberl (2010) proposed three methods to deal with potential rigidity – complete dynamic method, dynamic dimension correction method, and innovation practice method.

The key to the *complete dynamic method* is to maintain the rapid response of the enterprise’s core capacity to the changes in the market at any time. Then, the enterprise’s core capacity is dynamic to the market and can fully adapt to the sudden changes and risk factors in the market. When the “dynamic” core capacity of an enterprise is emphasized, some basic functions of the enterprise should also make a rapid response to changes and potential crises in the market, including human resources, finance, production, marketing, and strategic management, thus helping the enterprise re-optimize and reallocate its resources. Enterprises must grasp the information of market changes promptly and comprehensively, so as to form such a coping mechanism as makes objective, just and long-term judgments and interpretations of potential crises in the market.

The *dynamic dimension correction method* emphasizes that dynamic dimensions should be incorporated into the management process of the enterprise’s core capacity to ensure its relative flexibility. The core capacity can be adjusted from its orientation and development path or the process of its formation. Some relatively active indexes can be added to the core capacity that can be changed and adjusted timely in response to market changes, to ensure the stability of the mechanism of core capacity or further optimize it. The dynamic dimension correction method is often aimed at dealing with the core capacity that is already rigid or has shown a sign of rigidity. It emphasizes the activation of the dynamic capability index to the overall core capacity. Being a correction method in relatively late stages, it in fact generates a catfish effect.

The *innovation practice method* allocates a dynamic task to an established practice and helps the enterprise to overcome the rigidity trap of its core capacity through setting up practices of a unique type. After new practices are applied for several times, the loss that new practices bring to the enterprise must be taken into consideration. Thus, the application of new practices is often limited to areas with little impact. It takes a long time and lots of practices to implement the new practice in a key business and processing core event. Few enterprises will break their management practices to make decisions unless the enterprise has developed into a phase when reforms are needed.

### Influence of Cognition on the Development of Strategies and Competence in the Organization

Cognition is the basis for the development of organizational capabilities, which thus may affect the development process of capabilities, especially the cognition of what methods to adopt for dealing with potential rigidity. In existing researches, scholars have proposed two dimensions to understand managers’ cognition – cognitive structure and cognitive process. From the perspective of cognitive structure, managers’ cognition is the belief or mental model that a strategic decision-maker in the enterprise has, which reflects the state of the external environment, strategy, business portfolio, or the organization (Thomas and Porac, 2002). It is in essence reflected by a group of knowledge structure of the strategic decision-maker. From the perspective of cognitive process, managers’ cognition is the information processing for strategic decision-makers to understand the objective environment (Maynard, 1983). Decision-makers give feedbacks through scanning and explaining the information.

Since the 1990s, adhering to the “cognition–behavior” research paradigm, a large number of scholars have begun to adopt empirical research to explore the impact of managers’ cognition on the enterprise’s strategic behaviors. These behaviors include the construction of dynamic capabilities, the speed of strategic response, strategic changes, innovations, etc., and a growing number of studies have begun to further associate managers’ cognition with business performance, extending the traditional paradigm to “cognition–behavior–performance.” Emphasize the importance of dynamic management capabilities generated by the interaction between managers’ cognition and organizational capabilities to understand the strategic response of an enterprise facing the dynamic environment, namely the managers’ constructing, integrating, and reconstructing the resources, and the competence of an organization to help enterprises improve their individual ability to adapt to a dynamic environment (Adner and Zemsky, 2003). Eggers and Kaplan (2009) found that the interactions of managers’ cognition and organizational configuration positioning have exerted a significant influence on the updates of enterprise strategies, which indicates that when CEO focuses his or her attention on innovative technology, the positive role of organizational configuration positioning is enhanced for marching into the market in innovative industries. Reveal in their case study that managers’ cognition has a crucial influence on the evolution of corporate strategic behavior and organizational capabilities.

## Construction of Theoretical Model

Being the main body during the management behavior process, managers are often people or groups with certain rights, responsibilities, and the corresponding management capabilities to engage in real management activities. This article defines managers as members, including the company’s chairman, CEO, and senior managers reporting directly to them. In the case of WS Co., managers specifically indicate Dr. S and his team. As Dr. S served as Chairman and CEO of Suntech from 2000 to 2013, to ensure the continuity of the research subjects, this thesis limits the time of the research cases from 2000 to 2013, only considering the impact of managers’ cognition during this period on core rigidity.

Starting from the perspective of cognitive process, this thesis defines managers’ recognition as the process of information processing in which managers understand and respond to phenomena or events in their environment. From the perspective of the theory of cognitive behavior, cognition and behavior are interactive. Cognition is the cause of behavior, while behavior manifests cognition, which means that correct cognition leads to correct behavior, while wrong perception results in wrong behavior. Another key concept studied in this paper is the core rigidity, which is a negative process for companies. Thus, the manager’s cognitive errors must be grasped to reflect the influence of his or her cognition on this negative process. Such cognitive errors are reflected as errors in behaviors in real actions, which lead to core rigidity. Considering the research process, this thesis makes small adjustments to the basic paradigm of “cognition–behavior–performance” for research on managers’ cognition, changing it into the research paradigm of “cognition–behavior–results.”

As the core capacity paradox foreshadows the rigidity of core capacity, its three forms can be regarded as the methods to identify core rigidity-path dependence, structural inertia, and commitment. Thus, if the behavior caused by the managers’ cognition leads to the core capacity paradox, it is also the cause of core rigidity. Managers’ behavior is reflected by whether they deal with potential rigidity, which is shown by whether they adopt three treatment methods for potential rigidity, including complete dynamic method, dynamic dimension correction method, and innovative management method.

Meanwhile, considering the impact of environmental changes on core rigidity, this thesis discusses the role of managers’ cognition on core rigidity in two environments with moderate dynamic and high velocity. A moderate dynamic environment is where changes continuously take place while often following a rough and predictable linear trajectory. In such an environment, the industrial structure is relatively stable and market boundaries are relatively clear. The market participants (including competitors, consumers, and resources) are basically determined. Thus, uncertain factors show little impact. When enterprises are in a high-velocity environment, market boundaries become vague and market participants are no longer determined. Thus, changes are not linear or predictable under the influence of other uncertain factors, including the government’s intervention and policy environment.

Based on the case study of WS Co., this paper explains the two paths of how organizational competence turns rigid and the role played by managers in this process, taking into account the impact of environmental change. The first path lies in the wrong approach adopted by managers when facing potential rigidity, which leads to core rigidity. The second path is when managers fail to adopt any treatment methods for potential rigidity due to some other reasons, which results in core rigidity as well. This thesis also explores the possible reasons in the second path.

## Research Method

### The Case Company

Research in this paper is mainly carried out through a case study. As a common qualitative research method, case study is particularly applicable to problems relating to “How” and “Why” (Yin, 1994). In essence, the case study always follows inductive logic, making it suitable for the deep research of some complicated and specific cases in reality so that the theoretical contributions contained in the cases can be discovered.

A single case study is adopted in this paper for an exploratory study of theories. Compared with an empirical study and a multi-case study with abundant samples and data, the core strength of a single case study is much more apparent, in which the analysis can be carried out in a more concentrated way through more abundant, specific and deeper information, so that the study better fits the theory (Berg, 2001). This paper mainly deals with how WS Co.’s capacity developed, and how its core gradually grew rigid. Therefore, the research in this paper belongs to the longitudinal study with an emphasis on the process, where it is necessary to make analyses and comparisons in terms of how the cognition of managers influenced their disposal of potential rigid events, their choice of methods, their ability paradox, and the consequent rigidity, etc., in different periods, and to discover the evolution laws hidden behind. Based on the abovementioned reasons, a single case study is used in this paper.

#### Case Selection

In this paper, WS Co. is selected as the object of case study due to its distinct “uniqueness” and “theoretical enlightenment,” which can be applied to theory exploration (Eisenhardt, 1989; Yin, 2003). Primarily, as the one-time leading figure in the photovoltaic industry, WS Co. was founded in 2000 and was listed in 2005, ranked second worldwide in 2007, and underwent bankruptcy reorganization in 2013. It developed like a roller coaster in those years, during which its core capacity grew rigid step-by-step, making it quite representative; besides, as an entrepreneurial company, WS Co. was typically manager-oriented, where major decisions in the company were all made by Dr. S and his management team. It is easy to exclude other interference factors and thus explore the influence of “managers’ cognition” on core rigidity. Therefore, we can see that WS Co. corresponds well with the topic of this paper; lastly, as a typical case, WS Co. is a favorite of many researchers, and different scholars have already conducted deep researches on it from various perspectives. Besides, each stage of its development has also received much attention from different fields, making it convenient to obtain the data from diversified sources.

#### Data Sources

The difficulty of the research lies in the collection of materials and data. First, WS Co. went through the bankruptcy reorganization in 2013 and has been dissolved now. Senior executives and employees have gone to different places, and Dr. S himself has settled in the United States. Therefore, it is difficult to interview him directly and deeply. Besides, because outsiders do not know much about the inside information, the difficulty in obtaining first-hand information also increases. In addition, as the major purpose of this paper is to analyze how cognitive “mistakes” made by Dr. S and his management team influenced the core rigidity of the company, it is also not easy for anyone to reopen the old wounds and admit his mistakes in the past, especially for Dr. S, who used to be the richest man in the world; thirdly, no books or research reports on WS Co. have received its formal recognition till now. Besides, WS Co., as the one-time focus of the world, was good at whipping up public opinions through media, and it is even more difficult to judge the authenticity of information and screen the information with different opinions being voiced continuously through various informal channels.

This research started from January 2016 and lasted for 22 months, during which the abundant research data were collected from various sources. Considering the principle that data in a case study should come from different sources so that the research can be trustworthy and valid, a “triangulation method” was predominantly used in the research, in which different means were taken to study each phenomenon, so that new discoveries could be made through the convergence and intersection of multiple data with the avoidance of negative effects caused by prejudice (Yin, 1994). Three major approaches, a semi-structured interview, the collection of second-hand sources, and follow-up researches, were used in this research for data collection to ensure the diversity of information sources and, therefore, the solidity of research foundation (Yin, 1989). Sources are shown in Table 1.

**TABLE 1:** Sources.

Material type	Major source	Original target audience of the material	Number
Announcements and annual reports	Company website	Investors and the capital market	20
Academic studies	Mainstream academic journals	Students and teachers in universities, researchers	108
News reports	TV stations, newspapers, websites, and self-made media	Information demanders, observers, and netizens	209
Interview materials	Interviewees		Time (min)
	Wang Chao, chief editor of PV-Tech		120–150
	Li Lei, well-known expert in studies on photovoltaic industry		150–250
	Sun Yue, who had done case studies on WS Co. before		90–110

A semi-structured interview is helpful in the collection of data and information, especially the distinct information, which would change constantly as time goes by Eisenhardt (1989). Mr. Wang, the Chief editor of PV-Tech, Mr. Li, a well-known expert in the study of photovoltaic industry, and Ms. Sun, who had done case studies on WS Co. earlier, were interviewed, along with several semi-structured interviews in depth. They shared the following similarities: they all studied the photovoltaic industry for a long time and were thus well informed in all aspects.

The collection of second-hand materials was divided into four sections. First, materials of the industry which WS Co. was in were collected. The ups and downs of WS Co. were accompanied by the rise and fall of the industry, as well as the orientations of national policies. With the help of experts in the industry as well as professional websites like website of photovoltaic industry department and national research network, etc., the basic data of the photovoltaic industry were obtained so as to specify industry background and status of this company. Second, based on the information of basic situation and development of WS Co. obtained from its official website and relevant reports in media, key events during its development were identified. Third, through a retrieval of academic literature related to WS Co. on CNKI, 9 master’s theses, 99 journal articles, and 209 news reports were found. Fourth, details revealing the “core capacity,” “core rigidity,” “cognition of managers,” etc., were gleaned from the annual reports, financial statements, materials of internal conferences, speeches made by senior managers, etc.

*Follow-up study*: constant attention has been paid to WS Co. since January 2016. Although it was purchased by Jiangsu Shun Feng Photoelectric Technology Co., Ltd. in 2013, current development and situation of WS Co. after the purchase still receive much attention, with news media, research institutes, etc., publishing articles and expressing opinions. Analyses of the future development of WS Co. contained in these sources can still verify the research effectively. In addition, the analysis of WS Co.’s opponents cannot be ignored. Development paths, capacity changes, the decisions made by senior managers, etc., of the opponents in the same period were also meaningful in this paper with regard to reference.

## Research Discoveries

Based on the decoding and analyses of first- and second-hand materials, this paper points out the phenomena during the development of WS Co., which might result in potential rigidity, and presents managers’ “cognition–behavior–result” of the phenomena in each period, as shown in Table 2. To further explain the action mechanism of managers’ cognition during the core rigidity of the company and to figure out its path of core rigidity, analyses are done from the following three perspectives: (1) How the core rigidity was influenced when managers adopted three solutions to deal with potential rigidity mistakenly; (2) What had influenced the cognition and further resulted in the core rigidity when the managers did not take any measure to deal with potential rigidity; and (3) Managers’ cognitive preferences of the formation of core rigidity under a proper dynamic environment and rapidly changing environment.

**TABLE 2:** Process of core rigidity of WS Co.: analysis and discussion.

Analysis section	Cognition	Behavior		Result	
		Analysis frame	Evidences	Performance	Explanation
P1: managers dealt with potential rigidity in the wrong way and caused core rigidity.	There was a heavy reliance on foreign countries in the raw material market.	Complete dynamization	(1) Signed a 10-year supply contract of silicon slice with MEMC (2006); (2) Signed contract with OCI to purchase poly-silicon at a high price (2011).	Path reliance	Followed the experience of success in the past to store raw materials, and failed to notice that the price in the whole poly-silicon market was declining.
				Structural inertia	Other silicon suppliers with better quality and lower price had no access to it, and the purchase cost was locked, with an exit mechanism of high cost, high profit and high loss.
				Resource commitment	It was trapped in the resource commitment, where the organization selection and flexibility were limited.
	The structure of management team was complicated.	Dynamic and dimensional revision	(1) Appointed foreign employees to important positions (2004); (2) Established the board of directors to improve the structure of management team (2005).	Path reliance	The realistic path for privately owned photovoltaic companies to survive.
				Structural inertia	The board of directors was deprived of its power and there were still mutual constraints in decision-making.
	Products were limited, and business gathered in the midstream of the industry chain.	Innovation of common practice	(1) Invested in the field of thin-film solar energy (2007); (2) Acquired an upstream company relating to silicon slices (2008).	Structural inertia	Lacked business foundation to support the development of new business.
				Resource commitment	It was trapped in capital commitment, which increased the financial burden and cost pressure.
P2: managers failed to cope with potential rigidity due to some other reasons and caused core rigidity.	With the soar of achievements, there was an imbalance between personal profits and collective profits.	Family interest	(1) Connected transaction with and delivered benefits to Huihuang Silicon Technology and Asia Silicon (2006); (2) Dr. S refused to make unlimited warranty with his personal assets (2012).	Resource commitment	Kept up appearances and failed to make any changes in the later stage. Constantly strengthened decision-making, and was trapped in resource commitment.
				Structural inertia	Continued to strengthen the current pattern of behavior when it was fully aware of its insolvency.
	Made sure that the construction of the fund project went on smoothly.	Managers’ oversight	GSF anti-warranty scandal (2008).	Structural inertia	Lacked risk control and failed to break through the original pattern.
	Enlarged the capacity and achieved the biggest scale.	Lack of risk awareness	Expanded the production of silicon slices rampantly when the double-counter policies were against it (2011).	Path reliance	Followed the experience of success in the past blindly and enlarged capacity.
				Structural inertia	The cost, profit and loss of the exit mechanism were high.
				Resource commitment	Permitted the company to conduct high spillover transactions, and damaged its reputation.
P3: managers’ cognitive preferences of the process of core rigidity in different environments.	Positive	Properly dynamic	Took measures to deal with potential rigidity.		
	Negative	Rapidly changing	Took no measures to deal with potential rigidity.		

### Influence of Managers’ Disposal of Potential Rigidity on Core Rigidity

Dr. S and his team attempted to deal with the phenomena, that is, potential rigidity, which might result in the core rigidity in WS Co. in three ways. However, owing to the wrong methods, the core capacity of WS Co. grew rigid in the end. When the company was in trouble, managers tried to find the solutions. Therefore, managers’ cognition was still positive. We can see from Table 2 that managers took one of the three measures to deal with the potential rigidity in some events, leaving the rest of the events undisposed. This section only analyzes the processes where measures were taken and explores the role managers’ cognition play in them.

#### Complete Dynamization

Phenomenon analysis: although the photovoltaic capacity in China has already ranked first in the world, most of the photovoltaic products in Mainland China are supplied to European and United States markets, having a small market share in China. According to the survey, “90% of the photovoltaic products produced in China are sold to foreign countries and only 10% of them are digested in domestic market.” As for WS Co., 99% of its products are sold to European and United States markets, as shown in Table 3. Since its establishment, WS Co. was dependent heavily on overseas market, “with both of its ends, materials and market, in reliance of foreign countries.” During the decade from 2002, when the first production line was put into operation, to 2011, over 90% of WS Co.’s photovoltaic components were sold overseas. With the cancellation of allowance and arrival of the “double-counter” policy, European and United States markets shrunk seriously and domestic companies were deeply trapped.

**TABLE 3:** Regional distribution of WS Co.’s sources of income from 2004 to 2011.

	2004	2005	2006	2007	2008	2009	2010	2011
Europe								
Germany	61.53	101.59	254.40	685.80	570.90	701.80	818.50	631.00
Spain	1.66	18.16	123.50	466.20	718.70	61.10	86.50	44.60
Italy	–	–	–	–	117.10	200.10	473.90	150.60
France	–	–	–	–	–	108.40	223.00	239.00
UEB	–	–	–	–	–	–	–	132.50
Others	13.03	41.54	43.80	43.70	86.50	182.10	315.80	232.10
Subtotal in Europe	76.22	161.29	421.70	1195.70	1493.20	1253.50	1917.70	1429.80
United States	0.04	1.73	20.40	86.70	142.70	160.40	443.30	723.70
South Africa	1.35	0.49	1.90	0.90	1.90	–	–	–
China	6.70	56.40	129.70	25.70	134.90	75.70	154.00	371.60
Australia	–	–	–	–	–	33.50	120.00	136.40
Japan	–	–	4.30	8.50	6.70	81.60	134.20	143.90
Others	0.97	6.10	20.90	30.80	144.10	88.60	132.70	341.20
Total	85.29	226.00	598.90	1348.30	1923.50	1693.30	2901.90	3146.60
Proportion of Europe (%)	89.37	71.37	70.41	88.68	77.63	74.03	66.08	45.44
Proportion of Europe and United States (%)	89.42	72.13	73.82	95.11	85.05	83.50	81.36	68.44

*Unit: million dollars.*

Confronted with such a dilemma, Dr. S increased the reserve of raw materials to take the predominance of resources in peer competition. He signed “take-or-pay” contracts with suppliers of raw materials at a fixed price in 2006 and 2011, respectively.

##### Signed a 10-Year Supply Contract of Silicon Slice With MEMC (2006)

In 2006, the silicon market was in short of supply. Because there were not any large silicon slice companies in China, SUNTECH had to buy a large amount of silicon slices overseas. At that time, the price of poly-silicon soared rapidly and even hit the summit of USD 500/kg; in the meantime, the price of silicon slices also rocketed, reaching USD 80/piece. Under such a background, WS Co. signed a 10-year supply agreement of silicon slices with MEMC in the United States at a very alluring price, that is, USD 40/piece. This was an exclusive agreement, which required MEMC to provide sufficient silicon slices according to WS Co.’s demand (Anonymity, 2017). However, in the end of 2008, global financial crisis broke out and the photovoltaic market changed a lot in the crisis. In 2009, the price of poly-silicon dropped to USD 50/kg and the price of silicon slices declined from USD 80/piece to USD 2.4/piece (Zhou, 2010). Subsequently, domestic poly-silicon companies, such as GCL and Jiangxi LDK, developed rapidly, and competition in the poly-silicon market became fiercer and fiercer, with the price hovering at the bottom, causing a great loss to many photovoltaic battery manufacturers who had signed long-term agreements. At the beginning of July 2014, SUNTECH had to terminate the 10-year purchase agreement in advance. The agreement was valid from 2006 to 2016, and therefore, WS Co. paid USD 0.212 billion as the liquidated damage. Facing a huge amount of liquidated damage, the company terminated the agreement with no hesitation because the price of silicon slices was at a high point when it signed the contract and even if it decided to break the contract and find another partner, it may well save a large amount of cost in the future (Ye, 2011).

##### Signed Contract With OCI to Purchase Poly-Silicon at a High Price (2011)

In July 2011, the bomb, which was planted by WS Co. 5 years ago, started to explode. As mentioned earlier, SUNTECH had signed a 10-year supply contract with the poly-silicon magnate overseas, MEMC, which regulated that WS Co. shall purchase poly-silicon at the price of USD 80/kg. However, in 2010, the price had shrunk to USD 40/kg. As a result, WS Co. was overwhelmed and had to terminate the contract, paying USD 0.212 billion as the liquidated damage. The mistake continued. At the beginning of 2011, WS Co. signed a new long-term supply contract with poly-silicon magnate in South Korea, OCI, which regulated that WS Co. shall purchase at a price no lower than USD 35/kg, while the price of poly-silicon in the market had fallen to USD 20/kg. According to the internal staff, in its current purchase structure, OCI occupied one-third of the total purchase, followed by several domestic companies. Therefore, WS Co. had to continue to purchase at a price almost two times as high as the price in the market, or to pay a huge amount of liquidated damage again.

In such a circumstance where the price of raw material fluctuated in line with market conditions, Dr. S attempted to get rid of resource shortage through complete dynamization but neglected the development pace of the industry and the risk accompanying it. Indeed, in the environmental background at that time, many other solar energy companies like Yingli, LDK, etc., had also signed such supply contracts with poly-silicon manufacturers, but the contract terms were mostly around 4–7 years. Signing a contract with a term of 10 years did not correspond with the commercial practice of a thriving and promising industry. In addition, despite the fact that many senior executives raised their objections 1 week before the contract was signed (Bangbangshuxiang, 2013), Dr. S insisted in his decision to sign the 10-year purchase contract with MEMC, trapping itself in a dilemma where the company had to purchase silicon slices at a fixed price for 10 years. The increase of raw material purchase and expansion of production led to a high input and low output, as well as the loss of its advantage in competition. Other suppliers of silicon slices with better quality and lower price had no access into it. The purchase was fixed, trapping the company in resource commitment and limiting its organization selection and flexibility.

Likewise, SUNTECH purchased poly-silicon at a high price in 2011, giving rise to an exit mechanism with high cost, high profit, and high loss. Managers led by Dr. S only noticed that it was possible for them to purchase raw materials at a low price if they signed the contract with OCI, so they followed their experience of success in the past and reserved raw materials, whereas they paid no attention to the trend that the price in the whole poly-silicon market was declining. Now, we can see that WS Co. had already developed their path reliance and failed to reallocate the resources. Consequently, the company was not capable of coordinating and managing the upstream enterprises during the organization and management effectively, which eventually resulted in core rigidity.

#### Dynamic and Dimensional Revision Method

Phenomenon analysis: from the management structure of WS Co. which had lasted for many years, the company mainly consisted of the three types of staff, staff related to governmental departments, international teams, and relation networks. When implementing the policy of the company, subordinates in WS Co. did not dare to report the problems to their leaders due to some special “relation network,” making it another drawback of internal management in WS Co. The special “triangular” layout allowed WS Co. to take the first chance in the competition against its opponents, but it was not suitable for a long-term competition. Therefore, Dr. S tried to make up for the defect by dynamic and dimensional revision, that is, by appointing foreign staff to important positions and establishing the board of directors.

##### Appointed Foreign Staff to Important Positions (2004)

SUNTECH had a complicated organizational structure, as well as an unsound and a chaotic internal management mechanism. The lack of a scientific decision-making mechanism and effective checks and balances had given rise to many errors when making major investment decisions. In October 2004, Dr. S determined the scheme of organizational structure adjustment in the senior executive meeting, and foreign staff were appointed to the core positions in SUNTECH. After that meeting, founders who had started the business with Dr. S left their position one after another, leaving the foreign staff who were unfamiliar with the domestic situation there. Subsequently, the foreign team gradually obtained the power to make decisions and manage capitals. As a result, there were frequent connected transactions, and a large amount of capitals were occupied by connected companies overseas. The operation of the company was out of control, and WS Co. was hollowing out, becoming a channel to transfer capitals from China to other countries.

##### Established the Board of Directors to Umprove the Team Structure (2005)

According to the regulations of New York Stock Exchange, SUNTECH, as an overseas issuer, could be exempted from relevant regulations on the board of directors in *Listed Company Manual*, yet the company established a standard governing system of the board of directors. The educational and industrial backgrounds of the directors in WS Co. are shown in Table 4. The board of directors was not only characterized by high degrees (masters and doctors) but also included Dr. S, chairman and CEO, Fu Chengyu who had worked in the oil industry for over 30 years, Julian R. Worley who had 25 years’ experience in audit, and Susan Wang who had been in the positions of financial and senior management for 25 years in high-tech companies, Ji Jingjia, an outstanding expert in the photovoltaic industry, Zhang Weiguo who had worked in trust fund and high risk industries for 20 years, etc. We can see from the above mentioned examples that the overall talent composition in WS Co. is comparatively comprehensive, including both financial experts and management elites, absorbing the ideas at home and abroad, and containing both insurance and risks. However, the problems needless of too much explanations are stated: why did decision errors occur frequently? Did each director in the management team have certain decision-making power and management power? How large was their power?

**TABLE 4:** Members of the board of directors in WS Co.

	2005	2006	2007–2009	2010	2011
Chairman CEO	Dr. S	Dr. S	Dr. S	Dr. S	Dr. S
Directors	Ji Jingjia	Ji Jingjia			David King
	Zhang Weiguo	Amy Yi Zhang	Amy Yi Zhang	Amy Yi Zhang	
Independent directors	Qiu Zhizhong	Qiu Zhizhong	Qiu Zhizhong	Qiu Zhizhong	Qiu Zhizhong
	Jason E. Maynard	Julian R. Worley	Julian R. Worley	Julian R. Worley	Julian R. Worley
	Fu Chengyun	Fu Chengyun	Jason E. Maynard		
	Songyi Zhang	Songyi Zhang	Songyi Zhang	Sunsan Wang	Sunsan Wang

We can see that although Dr. S made an attempt to improve the problems in management structures by dynamic and dimensional revision, he still ended up relying heavily on foreign staff in terms of decision-making due to his neglect of structural inertia which the organization would fall into when important positions were given to foreign staff. As a result, the organization and management process grew rigid ultimately. On the top of that, WS Co. tried to avoid the relative defects in team composition by formulating a board system with high standards as well as strict membership access, yet it did not turn out well. After the board of directors was deprived of its power, WS Co. had fallen into an organizational and a structural rigidity of internal positioning. Being influenced by the promise of a high salary, key management position, international management patterns, etc., foreign senior executives made easy judgments in major issues, approved of the decisions carelessly, and even supported the connected transactions of WS Co., etc.

#### Innovation of Common Practice

Phenomenon analysis: major industry chains from the upstream to the downstream in the photovoltaic industry included poly-silicon, silicon slides, battery sheets, battery components, and solar photovoltaic power generation, while WS Co.’s business mainly focused on the manufacturing of solar cells and battery components in the midstream of the industry chain, with its products concentrated in crystal silicon battery. Accordingly, Dr. S was aimed at vertical integration and multiple business.

##### Invested in Thin-Film Solar Energy (2007)

Dr. S, a man with the doctorate of solar energy, once built WS Co., which produced crystal silicon batteries, into the largest photovoltaic battery manufacturer in the world. Nowadays, the photovoltaic industry is mainly divided into two camps, crystal silicon and thin film. The conversion ratio of crystal silicon battery is basically two times as high as that of thin-film battery, while the cost is also much higher than that of thin-film battery, so both sides enjoy their advantages. Currently, crystal silicon batteries have occupied over 80% of the market, becoming the predominant power. However, Dr. S always had a preference for thin-film battery because this was what he majored in. With his promotion, WS Co. continued to expand the capacity of crystal silicon batteries at a high speed on one hand, and on the other hand, it set aside a large amount of capitals for the research and development of thin-film batteries, which were not the mainstream, as well as the construction of factories. Due to its diversified investment, WS Co. had failed to make ends meet, and thus fell into the trouble of cash flow.

In May 2007, SUNTECH announced that it would invest USD 0.3 billion in Shanghai Pujiang High-tech Park for the research and development of thin-film batteries. However, in August 2010, it shut down the production base of thin films in Shanghai and shifted its focus to poly-silicon. This caused a loss of USD 84.4 million, including the loss of decrease in value, USD 54.6 million, and the loss of future rent, USD 29.8 million. Additionally, the joint venture, Sichuan SUNTECH, was established by WS Co., Materials Science and Engineering Department and Solar Materials and Equipment Department of Sichuan University for the research and development of cadmium telluride thin-film batteries in 2009. In the third quarter of 2011, the company decided to stop its operation in Sichuan SUNTECH. We cannot know the specific loss in this program for sure from the public materials, but according to the media, the loss was no less than USD 0.1 billion. When developing cadmium telluride thin-film batteries in Sichuan, Dr. S also invested USD 0.3 billion in Shanghai to construct traditional factory of thin-film battery. According to his plan, the capacity of the factory in Shanghai would reach 400 MW by 2010. However, the conversion ratio of SUNTECH’s battery was only 7%, far lower than that of its United States counterparts, 11–12%. Consequently, after the first section of this project was completed in July 2009, the company halted it and reconstructed it into a factory of crystal silicon, with a reason that thin-film battery had a gloomy future while crystal silicon battery was in huge demand. WS Co. announced to invest USD 2.68 billion in it, and the ultimate capacity would reach 1,000 MW. Hundreds to millions of dollars were lost during these alterations.

The year 2010 was considered as “the most profitable year” for the photovoltaic industry, yet Dr. S made mistakes in two major investments due to his wrong estimation of the market, and the company, consequently, suffered from a great deficit in this most profitable year. Owing to the adjustment, the net loss of SUNTECH Electric Power was up to USD 0.174 billion in the second quarter of 2010. While losing the control of the investment in thin-film batteries, SUNTECH also lost the control of investment in crystal silicon. A staff who once worked in WS Co. said that he had lost touch with Dr. S ever since he quitted the job (Liu, 2012b), and he believed that most of the staff left SUNTECH with nothing to miss. Dr. S was too stubborn, and he seldom listened to other people’s advice. It was almost inevitable that SUNTECH would end up this way. “The assets of WS Co. were used up by uncontrolled, unplanned, and chaotic investments one after another.”

##### Acquired an Upstream Company of Silicon Slice (2008)

To achieve the vertical integration of industry, WS Co. acquired an upstream company of silicon slices in an attempt to integrate the materials and production and sale. In 2008, WS Co. bought the equity in Shunda Holdings at the price of USD 98.9 million and became the largest shareholder. Shunda Holdings focused on the manufacturing of single-silicon rods and slices, and had a poly-silicon factory in Suzhou, which would supply WS Co. with poly-silicon reliably and stably. To counterbalance foreign companies, WS Co. also invested in a silicon ingot company and found a silicon material for three silicon ingot companies in China by virtue of its industrial influence. These producers would, in return, provide silicon ingot to WS Co. stably in the long run, and in this way, various uncertainties in the industry development could be eliminated. Unfortunately, in the same year, the largest global financial crisis in the 20th century broke out. WS Co. lost the financial support from a good market environment and business basis during its investment and acquisition. With the general fall in the price of silicon slices, the price of the silicon slices produced by SUNTECH Electric Power, on the contrary, was even higher than that in the market due to the cost of acquisition. That is to say, the acquisition did not bring WS Co. any advantage; instead, it added financial burden and cost pressure.

Thus, we can see that Dr. S had tried to make up for the drawback that WS Co.’s products were limited and were gathered at the midstream of the industry chain by opening up a new field for development through the innovation of common practice, yet he ignored the overall trend of the industry when pursuing the thin film blindly. Eventually, the marginalization of research ability, backward technology, wrong judgment of the development trend had led to a failure. WS Co. had to pay a tuition of hundreds to millions of dollars. More seriously, these problems had eventually resulted in the core rigidity during the resource restructuring.

**FIGURE 1:**
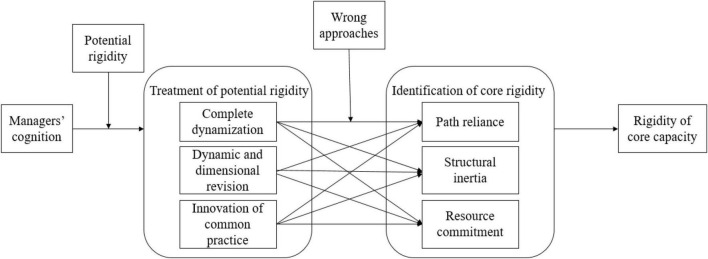
Path of core rigidity caused by managers’ wrong way of dealing with potential rigidity.

To summarize, in spite of the active measures that management team of WS Co. led by Dr. S took to solve a series of phenomena, which might lead to the core rigidity, they adopted the wrong approaches to handle rigidity, only to make the core capacity rigid at different levels. Thus, we can figure out the path of how managers’ cognition influenced core rigidity, as shown in Figure 1.

### Impacts on Core Rigidity When Managers Failed to Cope With Potential Rigidity

After the analysis of events in Table 2 to which measures were taken, what is going to be analyzed next is the potential rigidity to which WS Co. took no measures, along with the causes behind it. Besides, an analysis of how managers’ cognition led to the eventual core rigidity is also performed.

#### Family Interest

##### Connected Transaction With and Profit Transmission to Huihuang Silicon Technology and Asia Silicon Industry (2006)

In December 2005, WS Co. was successfully listed and raised a fund of USD 0.3 billion. Later, the company progressed smoothly, and its income had ranked high since 2005. In the end of 2006, Dr. S started to establish connections outside WS Co., and transmitted the profits to them through the connected transactions. According to the public materials disclosed by the company, “Asia Silicon Industry” and “Huihuang Silicon Technology” were both controlled by Dr. S in reality and belonged to the connections of WS Co.

###### Connected Transactions With Asia Silicon Industry (qinghai)

Being the actual controller of Asia Silicon Industry, Dr. S held close to 100% of its stocks. In 2009, Asia Silicon Industry sold 20% of its stocks to SUNTECH, and obtained a capital flow of USD 17.5 million. Later in 2010, WS Co. sold Asia Silicon Industry, earning nearly USD 4.1 million from the investment, and realizing USD 2,390. A scholar pointed out that: if WS Co. was related to Asia Silicon Industry only in terms of equity investment, it would not be harmed at all, unless the stock price of Asia Silicon Industry was substantially undervalued. However, Asia Silicon Industry did its business mainly with WS Co. during its existence, showing that behaviors hidden behind a series of connected transactions obviously did damage to the WS Co.’s shareholders’ rights and interests. In January 2007, Asia Silicon Industry signed a 16-year “take-or-pay” agreement at the price of USD 1.5 billion and paid USD 49.6 million in advance. In the later stage, WS Co. had to purchase poly-silicon at the price of USD 30/kg, while the price of poly-silicon in China was USD 26/kg. Calculating it by the ratio that the price in contract was 15% higher than that in the industry, WS Co. transferred USD 26.03 million to Asia Silicon Industry and Shi’s family.

###### Connected Transaction With Huihuang Silicon Technology (BIV)

According to a survey, the controlling owners of Huihuang Silicon Technology were Dr. S and his wife, Zhang Wei. The connected transactions between Huihuang Silicon Technology and WS Co. were mainly done during 2008 and 2011. WS Co. paid money to Huihuang Silicon Technology for several times to purchase and to acquire equity. For instance, in 2008, it paid USD 230.6 million in advance, and in 2009, USD 200.3 million; besides, there was a huge positive gap between the prepaid amount and actual amount. To purchase raw materials, WS Co. paid another USD 130 million to Huihuang Silicon Technology during 2008 and 2010, and the payment was never stopped, leading to the situation that a large amount of WS Co.’s capital flew to Huihuang Silicon Technology. Meanwhile, Huihuang Silicon Technology utilized it for free.

We can tell from the abovementioned data that profit transmission between WS Co. and its connected companies was not only featured by a large amount but also by the high frequency. As Dr. S was the controlling owner of each of its connected companies, one cannot help but suspect that Dr. S was transmitting profits to his family companies by taking advantage of his position. In terms of management, connection or the enhanced transactions between a company and its connected ones can control the external risks to some extent, and to control the quality of raw materials or semi-finished goods. From the perspective of cost reduction, a company can better integrate its resources on the whole through transactions with connected companies, and further optimize its industry chain, expand its business range, and gain profits from equity transaction. Of course, a company can obtain floating capitals by equity transaction and achieve the win–win or even multi-win state between itself and its connected companies (Du, 2015). Nonetheless, it is obvious that the transactions among WS Co. and Asia Silicon Industry and Huihuang Silicon Technology never achieved the win–win state as WS Co. never dealt with the purchase of poly-silicon or raw materials with equity and justness, and too much “help” it offered to the connected companies had damaged the stockholders’ interests and rights directly.

It is possible that, at first, Dr. S worked for the win–win situation with an intention to control external risks and reduce the cost, yet lured by his irrationality, he gradually lost control of it and continued to make profits by taking advantage of his position, so when he was investigated in the later period, he had to keep up appearances and continue to transmit profits to his connected companies, plunging WS Co. into resource commitment, where the resource positioning of the company changed due to the loss of internal resources and finance, and eventually, the connected companies lost their profits.

##### Dr. S Refused to Make Unlimited Liability Guarantee With his Personal Assets (2012)

Government had offered Dr. S the chance to withdraw, that is, to sell his equity in WS Co., which would be taken over by Guolian; in the meantime, China Development Bank would continue to offer loan to WS Co., on the condition that Dr. S shall make unlimited liability guarantee with his personal assets. However, he refused (Liu, 2012a).

A person who left WS Co. said that, “In fact, WS Co. was only part of Dr. S’s personal assets (Zhong, 2013).” This person also believed that if the company was to be re-organized, the connected transactions with Asia Silicon Industry and other companies would definitely be exposed when the government check its accounts as well as some bad debt, which were unknown by the public. Therefore, fearing that his personal reputation might get damaged and that the connected transactions would be exposed, Dr. S declined the “remedy” offered by Wuxi government. Besides, being fully aware of the insolvency of the company, he refused to make any guarantee resolutely and left the company sinking or swimming on its own. He could also shrink from his responsibilities by bankruptcy, maintaining the reputation of the company in a reasonable and legal way, trapping the company capacity within the internal reputation mechanism.

In summary, Dr. S chose personal interest over a collective one when there was an antagonism between the two. “Many senior executors were fully aware of Dr. S’s accumulation of personal wealth, they just turned a blind eye to it.” The insider also said, “when Asia Silicon Industry was at its initial stage, colleagues in SUNTECH were asked to strive for the financial support from banks and to make road shows for it. In such a circumstance, was it possible that SUNTECH Electric Power was totally ignorant of it?” In reality, after Wuxi government reorganized the assets in SUNTECH Electric Power and withdrew the national capital safely from the company, WS Co. had already become a privately owned company in 2005. In 2006, with a series of Dr. S’s efforts to establish the connected companies (which were indeed Shi’s family companies) and transmit profits, WS Co. was fully qualified as a listed company, as well as a family company. When Chandler first defined family company, he believed that if two powers (management right and ownership) in a company were integrated together, the company was qualified as a family company (Chandler, 2002). Connelly suggests that a family company should be managed by at least two generations, and the profits and targets it conveys should affect each other. Besides, a family company has to meet seven conditions, including the integration of company value and family value. Jeff offers a simpler definition, that is, a company owned and controlled by one or several family members is a family company. In such a “family company,” it was difficult for Dr. S to distinguish the company interest from family interest fairly, and therefore, he gradually erred on the side of family interest.

#### Oversight of Managers

GSF anti-warranty scandal (2008). This event took place in May 2010 when WS Co. provided guarantee for the loan, around EUR 554.2, provided by the National Development Bank to Solar Puglia II, S.ar.L (the investment company of GSF). Besides, WS Co. was also required to deposit cash, as an installment, in a commercial bank in Luxembourg to mortgage around EUR 30 million. At the same time, GSF Capital Pte Ltd., a parent company of GSF, provided counter guarantee with EUR 560 million of German government bond for WS Co. (Anonymity, 2012). In July 2012, WS Co. planned to realize its investment in GSF, and therefore it hired an external consultant to evaluate the whole process, in which it discovered that the bond might not exist, and the company would thus become the victim. So, what was the relation between WS Co. and GSF? Why WS Co., a leading company in the industry, could be “deceived” by GSF? How would it damage WS Co.?

**FIGURE 2:**
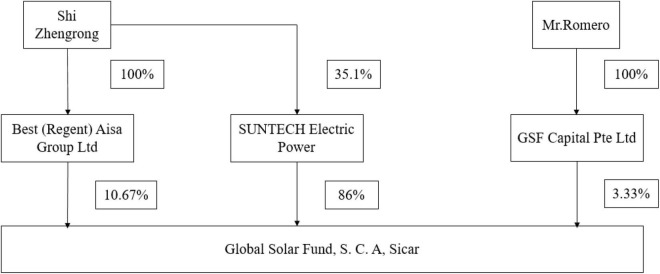
Stock structure of GSF in 2008.

In February 2008, GSF was founded in Luxembourg, focusing on the investment in the development, construction, and operation of solar photovoltaic power station in Europe. WS Co. was its major investor. WS Co.’s annual report in 2008 disclosed that the company had decided to continue its strategic investment in photovoltaic companies, including GSF. WS Co. invested EUR 258 million in total, taking up 86% of GSF’s stocks; Dr. S also had 10.67% of GSF’s stocks through Best (Regent) Aisa Group Ltd. Being the limited partner (LP) of GSF, WS Co. had 50% of the vote; being the general partner of GSF, GSF Capital Pte Ltd. held 3.33% of GSF’s stocks through its branch, and was in charge of it. Mr. Romero was the ultimate controller of GSF Capital Pte Ltd. Chart of GSF’s stock structure in 2008 as shown in Figure 2.

From 2009 to 2011, WS Co. sold photovoltaic components to the power station project invested by GSF, making a profit of around USD 350 million. In 2010 and 2011, the program invested by GSF completed the construction projects with 105 and 40 MW, respectively, and was thus re-evaluated and appreciated by the investment company through a fair value, bringing a high investment profit for WS Co. In addition, WS Co. promised to invest another EUR 258 million to GSF, and by the end of 2011, it had invested EUR 156 million. In July 2012, WS Co. discovered that the German government bond, which was EUR 560 million, did not exist at all when it planned to realize the investment in GSF. Eventually, WS Co. lost RMB 5.96 billion in the GSF program (Ye, 2013).

It can be seen from Figure 2 that GSF was a connected company of WS Co. in essence, and Dr. S had over 40% of GSF’s stocks through Best (Regent) Aisa Group Ltd. and WS Co. (100 × 10.67% + 35.1 × 86% = 40.856%). Besides, Mr. Romero, the ultimate controller and Class A manager of GSF, once worked for WS Co. Therefore, GSF was highly related to WS Co. in terms of both stocks and senior executives. Under such a background, WS Co. failed to authenticate the bonds, which could be verified with a simple investigation in advance. Senior executives in WS Co. were surely to blame for the collective oversight. The oversight of managers refers to the case where Dr. S and his management team in SUNTECH Electric Power lacked a clear understanding of management decisions and decision results, failed to pay enough attention to a wrong decision, and thus made serious mistakes one after another. Facing warranty projects where each side should have a thorough understanding of the other, they still lack the awareness of risk control, and were unable to get rid of the inertia of original risk control structure. The loss of internal resources and finance eventually led to the rigidity in its resource positioning.

#### Lack of Risk Awareness

The production of silicon slices expanded recklessly when the double-counter policies were against it (2011). When the sign of overcapacity started to show up in the photovoltaic industry in China, many companies in the photovoltaic industry, represented by WS Co., still insisted on the idea that there was not any overcapacity in this industry. The whole industry continued to expand the capacity due to this cognition. In 2011, the capacity of photovoltaic components in China was around 40 million kW, while the components being installed in the whole world was only 28 million kW. Photovoltaic capacity in China had surpassed the demand of the whole world over a decade. Being influenced by European debt crisis, European market, from which almost 80% of the world’s photovoltaic demand came, shrunk sharply; in addition, United States had implemented the double-counter policy against Chinese photovoltaic products. Both factors had given rise to a sharp decrease in the orders in Chinese photovoltaic companies, aggravating the low price competition and damaging the whole industry.

In 2011, SUNTECH expanded the production of silicon slice recklessly through Rongde New Energy Ltd., a wholly owned subsidiary, enlarging the capacity from 500 to 1,600 MW in a year. In 2011 alone, the capital expense of SUNTECH was USD 366.8, mainly on the expansion of silicon slice capacity in Rongde. In that same year, the shipment was 2,066 MW, increasing by 34% compared to that in 2010; however, its price of battery sheets dropped from USD 1.43/W to USD 0.46/W, and the price of components, from USD 1.82/W to USD 1.51/W, reducing its gross margin to 12.3%, the ever lowest figure. Therefore, the irrational expansion of a company can lead to the excessive overcapacity, inability to adjust to the changes in the International market, universal difficulty in operation, and the breakage of part of the capital chain. This was the major cause why WS Co. went bankruptcy and why the photovoltaic industry in China was troubled.

It can be concluded that Dr. S, to make up for the wrong decision he made in the past, did not fully understand the prospect of the market, lacked risk awareness, and stuck rigidly to the idea that there was no overcapacity in the photovoltaic industry in China. He expanded the production of raw materials when the market was not on his side, followed the old path that “those who have the largest scale win the game” and tried to grab the market share. All these measures deepened SUNTECH’s path reliance, and eventually generated process rigidity. Risk awareness refers to the feelings and understandings of risks, as well as a company’s judgment of the market and technology innovation based on the relation between company’s interest and risk. Yet, due to managers’ lack of risk awareness, the company expanded the production of silicon slices in 2012 when there was a nose dive in the price of battery sheets. This wrong decision did not only leave a large quantity of silicon slices gaining dust in the warehouse but also caused the low-price amortization and even the overstock of silicon slices. As a result, Dr. S permitted the company to conduct high spillover transactions and ultimately damaged its own reputation.

**FIGURE 3:**
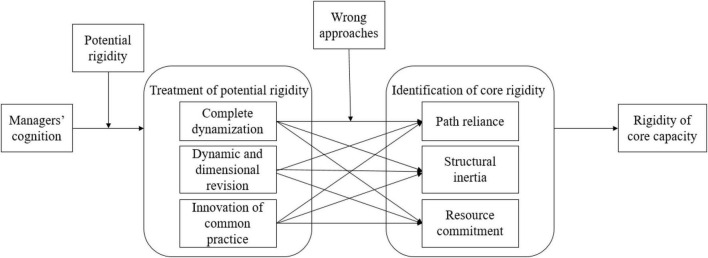
Path of core rigidity caused by managers’ failure of solving potential rigidity.

In summary, the management team led by Dr. S in WS Co. failed to take the corresponding measures to deal with the potential rigidity owing to family interest, manager oversight, and the lack of risk awareness, and therefore caused the capacity rigidity. The path of how managers’ cognition influenced core rigidity can be figured out as shown in Figure 3.

### Preferences of Influence of Managers’ Cognition on Core Rigidity in Different Environments

Through the observation of the timeline in the two paths of influence of managers’ cognition on core rigidity, it is discovered that managers’ cognitive preference may differ in different environments.

When the outside environment was moderately dynamic (2001–2008), managers could take the corresponding measures to deal with the potential rigidity; for instance, it signed a silicon slice supply contract with MEMC for 10 years (2006), established the board of directors to improve the structure of management team (2005), invested in the thin-film solar energy field (2007), acquired its upstream silicon slice companies (2008), etc. In this period, the photovoltaic industry rose steadily, supported by the stable market and policies, and the output, as well as the number of photovoltaic companies being listed, increased every year. Thus, it can be summarized that when the outside environment was moderately dynamic (2001–2008), it was easier for managers to deal with rigidity, even if the measures they took were inappropriate. That is to say, a stable environment is more likely to arouse managers’ passion of reform.

When the environment was fast changing (2008–2013), managers failed to take effective measures to deal with potential rigidity, and thus deepened the core rigidity, such as GSF counter warranty scandal (2008), rampant expansion of silicon slice under the unfavorable double-counter policy (2011), and Dr. S’s refusal of making unlimited liability guarantee with his personal assets. At that time, the photovoltaic industry was undergoing an unrest, with the outbreak of financial crisis, the sharply decreasing demand in European and United States markets caused by the “double-counter” policy, overcapacity in the photovoltaic industry, and the closing down of companies. Thus, it can be summarized that, when the outside environment was changing rapidly (2008–2013), managers were influenced by other factors unlikely to deal with potential rigidity and would unavoidably cause core rigidity.

**FIGURE 4:**
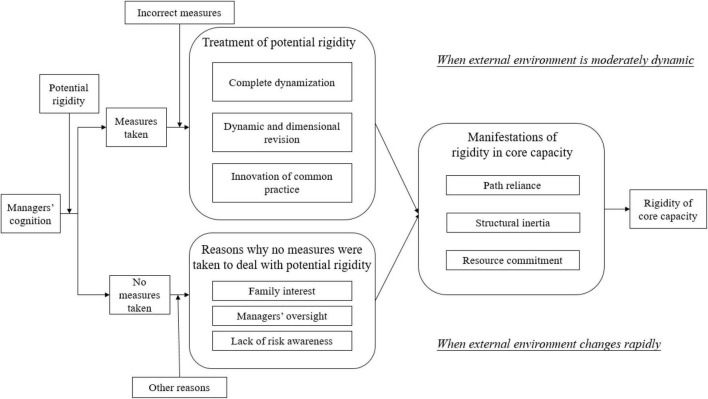
Model of preferences of influences on core rigidity caused by managers’ cognition in different environments.

Therefore, we can summarize the preferences of influences of managers’ cognition on core rigidity in different environments as shown in Figure 4.

## Conclusion and Significance

To conclude, this paper clearly explains how core capacity grows rigidly step-by-step through the interaction between managers’ cognition and treatment of potential rigidity, especially managers’ cognition, one of the reasons behind the formation of core rigidity. It helps managers to re-examine the core capacity of a company, to re-check their decisions made during the company’s development, and to amend rigid capacity and their wrong cognitions, so that the companies will develop in a better way.

Through the case study, WS Co., this paper has pointed out the phenomena that are likely to give rise to the rigidity in the core capacity of a company, figured out the “cognition–behavior–result” process of these them as well as the mutual effects, and discovered that managers’ cognition plays an important role in the formation of core rigidity. Theories related to core rigidity, including core capacity, core rigidity, treatment of potential rigidity, managers’ cognition, etc., have been applied in this paper to the analyses of WS Co.’s development and its cognitive process. This paper thus believes, through analyses, that the core rigidity in WS Co. is characterized by resource commitment, structural inertia, path reliance, etc. Besides, cognitions and decisions of the management team led by Dr. S had speeded up its bankruptcy and reorganization. When potential rigidity occurred in each period, managers and the team, influenced by their cognitive mistakes, took the wrong actions to deal with it or even failed to deal with it, and therefore led to the core rigidity. Specific conclusions are as follows.

Theoretical implications follow. First, this study extends recent literature studies on the risk-based assessment of a firm’s core rigidity emergence (e.g., Wang et al., 2021), by investigating the dynamics of post-risk assessment decision influences. Such an extension demonstrates the impact of realized decision-making biases after entrepreneurs have “seen” the potential risks. Primarily, when rigidity occurs, managers and management team of a company have positive cognitions and take measures to deal with the potential rigidity, yet the incorrect approaches they have chosen lead to the ultimate core rigidity.

Second, this paper also extends the above risk-decision nexus to an action aspect, implicating a complete risk-decision-action framework for the emergence and impacts of core rigidity. The value of a firm’s capability should be able to be realized exactly (Eastburn, 2018). Action is one of the necessary condition for such realization. Especially, as we demonstrated with the case study, when potential rigidity occurs, managers and management team have negative cognitions but fail to deal with it with effective actions due to some other factors, such as family interest, managers’ oversight, and the lack of risk awareness (e.g., Olaleyea et al., 2020) – eventually leading to core rigidity.

Lastly, this paper extends the literature of cognition-core rigidity relations by showing that managers’ preferences of cognition vary in different situations, bringing a contingency perspective into the discussions. When the outside environment is moderately dynamic, managers are likely to have positive cognitions and take measures to deal with rigidity. Ironically, however, even if the measures are incorrect, they still reveal that a stable environment is more likely to arouse managers’ passion of reform. In contrast, when the outside environment is rapidly changing, managers tend to have negative cognition and fail to deal with potential rigidity due to the influence of other factors, and finally cause core rigidity. This implicates that, when business environment is in dynamism, managerial responses to core rigidity and their following measures to rigidity could either fall in a virtuous or negative cycle, given no other decisive influences on the single or few decision-makers at the top.

Practically, implicated by the abovementioned theoretical insights, we suggest that firms, especially those in dynamic environment and operationally and decisively held tightly by single or few influential persons (i.e., entrepreneurs, TMT member, and key stakeholders), should build up co-governance mechanisms, which allow a continuous and collective reflection on key assessment, decisions, and actions toward perceived core rigidity. This also applies to core capability management because in dynamism core capability could turn into potential rigidity at any moment. Furthermore, we suggested that new emergent technologies such as big data or artificial intelligence could be adopted to assist human-making assessment, decision, and solution choices for the capability-rigidity issues, to make the best use of firm’s collective intelligence and knowledge bases.

## Data Availability Statement

The raw data supporting the conclusions of this article will be made available by the authors, without undue reservation.

## Ethics Statement

The studies involving human participants were reviewed and approved by the Tianjin University. The patients/participants provided their written informed consent to participate in this study.

## Author Contributions

YG conceived and designed the research and provided guidance throughout the entire research process. YG and MC wrote and supplemented the English manuscript. P-WH performed the data processing and analyses. S-CF and F-ST reviewed and edited the manuscript and were responsible for all R&R works. All authors contributed to the article and approved the submitted version.

## Conflict of Interest

The authors declare that the research was conducted in the absence of any commercial or financial relationships that could be construed as a potential conflict of interest.

## Publisher’s Note

All claims expressed in this article are solely those of the authors and do not necessarily represent those of their affiliated organizations, or those of the publisher, the editors and the reviewers. Any product that may be evaluated in this article, or claim that may be made by its manufacturer, is not guaranteed or endorsed by the publisher.

## References

[B1] Adner R. Zemsky P. (2003). A Demand-Based View of Sustainable Competitive Advantage: The Evolution of Substitution Threats, Resource rents and Competitive Positions. Unpublished Working Paper Insead, 2003. 10.2139/ssrn.651184

[B2] Anonymity (2012). *Suntech’s GSF Trap: Who is Lying on Earth? Global Entrepreneurs.* Available online at: http://www.china5e.com/news/news-239093-0.html (accessed October 24, 2017).

[B3] Anonymity (2017). *Wuxi Suntech Payment of 212 Million Dollars in liquidated Damages.* Available online at: https://www.aliyun.com/zixun/content/2_6_174405.html (accessed November 24, 2017).

[B4] Bangbangshuxiang (2013). *The Life and Death Crisis of Suntech.* Available online at: http://solar.ofweek.com/2013-01/ART-260008-8500-28663553.html (accessed October 08, 2017).

[B5] Berg B. L. (2001). *Qualitative Research Methods for the Social Science.* Boston, MA: Allyn and Bacon.

[B6] Bettis R. A. Prahalad C. K. (1995). The dominant logic: retrospective and extension. *Strategic Manag. J.* 16 5–14. 10.1002/smj.4250160104

[B7] Chandler A. D. (2002). *Strategy and Structure: Several Chapters of the Growth of American Industrial and Commercial Enterprises.* Yunnan: Yunnan People’s Publishing House.

[B8] Du Y. (2015). A brief analysis of the impact of related party transactions on enterprises. *Finance Econ. Acad. Edn.* 30 54–54.

[B9] Eastburn R. (2018). Realising value from absorptive capacity. *J. Inform. Knowledge Manag.* 17:1850011. 10.1142/S0219649218500119

[B10] Eggers J. P. Kaplan S. (2009). Cognition and renewal: comparing CEO and organizational effects on incumbent adaptation to technical change. *Organ. Sci.* 20 461–477. 10.1287/orsc.1080.0401 19642375

[B11] Eisenhardt K. M. (1989). Building theories from case study research. *Acad. Manag. Rev.* 14 532–550. 10.2307/258557

[B12] Helfat C. E. Peteraf M. A. (2015). Managerial cognitive capabilities and the microfoundations of dynamic capabilities. *Strategic Manag. J.* 36 831–850. 10.1002/smj.2247

[B13] Leonard-Barton D. (1992). Core capabilities and core rigidities: a paradox in managing new product development. *Strategic Manag. J.* 13 111–125. 10.1002/smj.4250131009

[B14] Levinthal D. A. March J. G. (1993). The myopia of learning. *Strategic Manag. J.* 14 95–112. 10.1002/smj.4250141009

[B15] Liu X. (2012a). *Shi Zhengrong was Trapped in the Besieged city of Wuxi When SUNTECH Went Bankrupt.* Available online at: http://www.cb.com.cn/deep/2012_1020/420806.html. (accessed October 24, 2017).

[B16] Liu X. (2012b). *The Open Contradiction Between Shi Zhengrong and the Government of Wuxi.* Available online at: http://jjckb.xinhuanet.com/2012-10/22/content_407595.htm. (accessed October 24, 2017).

[B19] Maynard M. (1983). *Environment, Natural Systems, and Development: An Economic Valuation Guide.* Washington D. C: The Johns Hopkins University Press.

[B20] Olaleyea B. R. Akkayab M. Emeagwalic O. L. Awwadd R. I. Hamdane S. (2020). Strategic thinking and innovation performance; the mediating role of absorptive capabilities. *Rev. Argentina Clín. Psicol.* XXIX, 2030–2043.

[B21] Prahalad C. K. Hamel G. (1990). *The Core Competency of the Corporation.* Boston, MA: Harvard Business Review, 792–799.

[B22] Schreyögg G. Kliesch-Eberl M. (2010). How dynamic can organizational capabilities be? towards a dual-process model of capability dynamization. *Strategic Manag. J.* 28 913–933. 10.1002/smj.613

[B24] Teece D. J. Pisano G. Shuen A. (1997). *Firm Capabilities, Resources and the Concept of Strategy.* Berkeley, CA: University of California.

[B25] Thomas H. Porac J. F. (2002). Managing cognition and strategy. *Issues Trends Future Direct.* 2002 638–656.

[B26] Wang H. Tian M. Zhang Y. Wang Z. (2021). “The impact of R&D strategy on firm performance of ICT companies in China,” in *Management for Sustainable and Inclusive Development in a Transforming Asia*, eds Shioji H. Adhikari D. R. Yoshino F. Hayashi T. (Singapore: Springer), 10.1007/978-981-15-8195-3_13

[B27] Ye W. (2011). *Suntech Compensation for Danger and Machine Behind $200 Million.* Available online at: http://roll.sohu.com/20110725/n314410858.shtml (accessed October 8, 2017).

[B28] Ye W. (2013). *Wuxi Suntech is Deeply Troubled by Internal and External Troubles: Difficulties in Overseas Sales and Difficulties in Domestic Survival Chinese Business Newspaper.* Available online at: http://finance.ifeng.com/a/20130928/10777028_0.shtml (accessed October 24, 2017).

[B29] Yin R. (1994). *Case Study Research: Design and Methods.* Thousand Oaks, CA: sage.

[B30] Yin R. K. (1989). *Case Study Research Design and Methods.* Hoboken, NJ: Blackwell Science Ltd.

[B31] Yin R. K. (2003). Case study research design and methods applied social research methods series. *J. Adv. Nurs.* 5 108–108. 10.1046/j.1365-2648.2003.02790_1.x

[B32] Zhong J. (2013). *Shi Zhengrong Wealth Seven Years Evaporation 18 Billion 600 Million Analysis said Shi Zhengrong is Still Invisible Rich.* Available online at: http://finance.ifeng.com/news/special/wxsddl/20130322/7807148.shtml. (accessed October 24, 2017).

[B34] Zhou Q. (2010). China’s photovoltaic industry both sides suffer. *Chinese Machinery* 18 2–9.

